# Abatacept Treatment Does Not Preserve Renal Function in the Streptozocin-Induced Model of Diabetic Nephropathy

**DOI:** 10.1371/journal.pone.0152315

**Published:** 2016-04-07

**Authors:** Jenny Norlin, Lisbeth Nielsen Fink, Peter Helding Kvist, Elisabeth Douglas Galsgaard, Ken Coppieters

**Affiliations:** Novo Nordisk A/S, Måløv, Denmark; University of Houston, UNITED STATES

## Abstract

Diabetic nephropathy (DN) is one of the most severe complications of diabetes and remains the largest cause of end-stage renal disease in the Western world. Treatment options are limited and novel therapies that effectively slow disease progression are warranted. Previous work suggested that treatment with CTLA4-Ig (abatacept), a molecule that binds and blocks B7-1 and is licensed for the treatment of rheumatoid arthritis, could ameliorate DN. This study was designed to assess whether B7-1 signalling constitutes a promising therapeutic pathway for DN. Mice injected with streptozotocin (STZ) were treated with abatacept and glycemia and renal function were assessed. No differences were found in diabetes progression, albumin excretion rates or albumin/creatine ratios, while mesangial expansion was unaltered at endpoint. Except for increased renal CCL5, treatment did not affect a panel of gene expression endpoints reflecting early fibrotic changes, inflammation and kidney injury. Finally, abatacept treatment effectively reduced the accumulation of activated CD4^+^ T cells in the kidney, suggesting that renal T cell inflammation is not a driving factor in the pathology of the STZ model. In conjunction with the recent data discounting the expression of B7-1 on podocytes, our present data do not support a role for abatacept in DN treatment.

## Introduction

Diabetic nephropathy (DN) is clinically defined as the progressive development of renal insufficiency in the setting of hyperglycaemia and it is one of the most severe complications of diabetes. It remains the single largest cause of end-stage renal disease in the Western world, having a devastating impact on patients with diabetes and placing a major burden on health care resources [1]. There is therefore an urgent need for novel therapies to be developed to inhibit the progression of the disease.

There is mounting evidence that existing drugs developed to treat other metabolic, inflammatory, and immunological disorders, may also be effective in preventing or slowing the progression of DN to end stage renal disease [2]. Podocytes reportedly express CD80 (B7-1) in response to LPS and other types of stress, and lack of this molecule was found to significantly reduce LPS-mediated podocyte injury [3]. Moreover, treatment with CTLA4-Ig (abatacept), a molecule that binds B7-1 (and, with lower avidity, B7.2), blocks B7-1 on podocytes and is licensed for the treatment of rheumatoid arthritis, reduces urinary albumin excretion and improves kidney pathology in two animal models of DN [4]. Fiorina et al showed that CTLA4-Ig acts by preventing the B7-1-induced cytoskeletal rearrangements leading to podocyte detachment and loss. Since peripheral cytokine levels were found unaltered upon treatment, its therapeutic effect was ascribed to a podocyte-specific protective mechanism. A recent study by Gagliardini et al, however, failed to demonstrate B7–1 expression in human and experimental DN, casting doubt on the applicability of abatacept, targeting podocyte B7–1, for the prevention or treatment of DN [5].

CTLA4-mediated co-inhibitory signalling is essential in the regulation of T cell immunity, with a non-redundant role in controlling T cell priming [6]. However, the role of activated T lymphocytes in the pathogenesis of DN is still unclear. Elevated numbers of activated effector T cells are found in kidneys of the streptozotocin (STZ) induced mouse model for DN, as well as in those of DN patients [7]. The mechanisms underlying their recruitment and the consequences of their influx into the kidney are only partially understood [8]. Given that podocytes are independently capable of inducing T cell proliferation, differentiation and effector function, it is hypothesized that they could actively contribute to tissue destruction by T cells [9].

The objective of the present study was threefold. First, we wanted to reproduce the data by Fiorina et al in the STZ mouse model to assess whether B7-1 signalling constitutes a promising therapeutic pathway for DN. Second, we intended to establish abatacept as a positive control treatment for future screening of T cell-targeted drugs for DN in the STZ model. Finally, we focused on the impact of co-stimulation blockade on T cell influx and activation state in the kidney to evaluate whether T cells are causal to renal injury in the STZ model.

## Results

Male 129Sv mice injected with streptozotocin developed hyperglycemia and increased HbA1c as expected [10], accompanied by weight loss. Treatment with abatacept or vehicle did not affect any of these parameters and there were no baseline differences between the two diabetic groups (S1 Fig).

**Fig 1:**
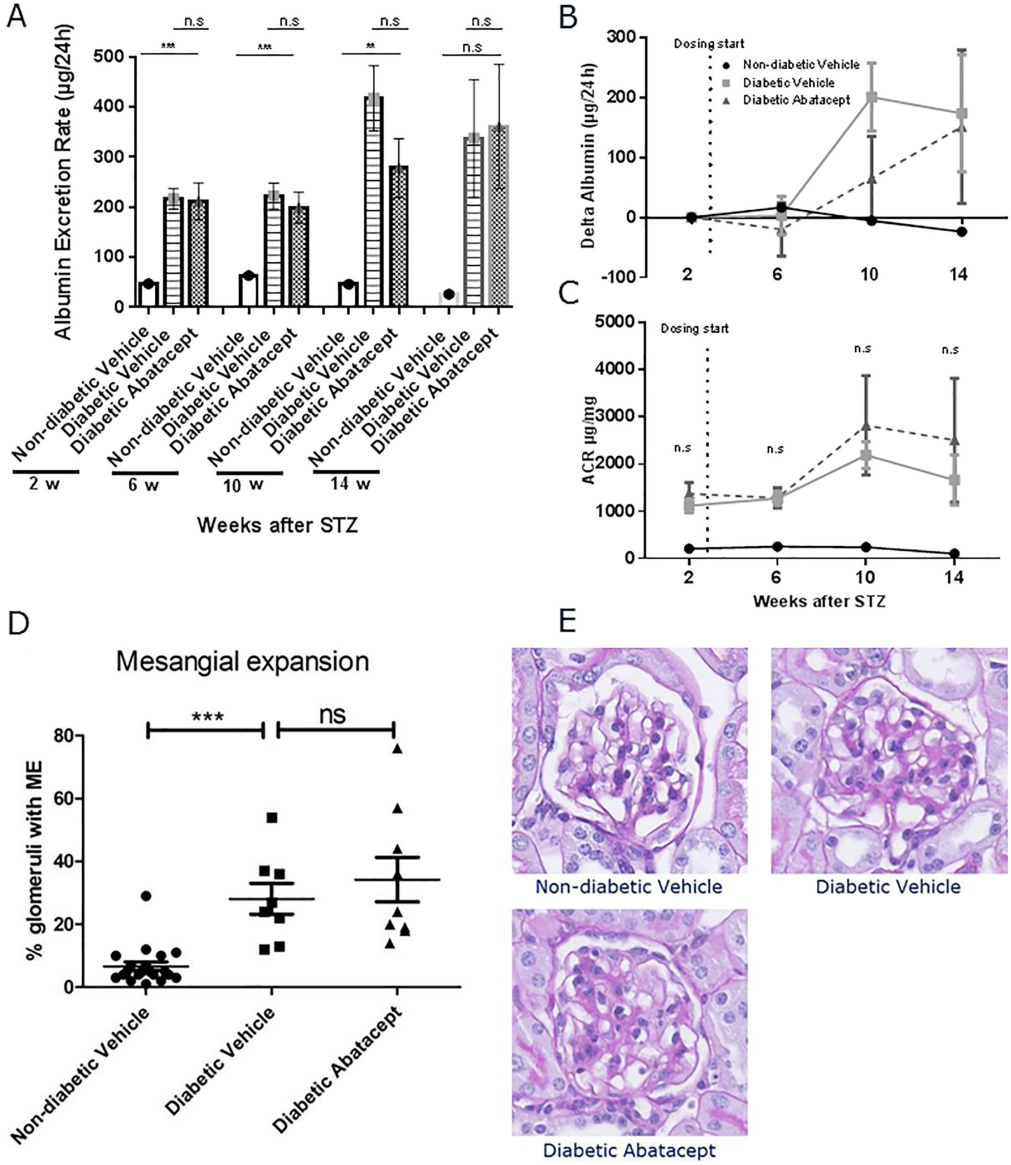
Effect of abatacept treatment on the development of albuminuria and mesangial expansion. A) 24h urinary albumin excretion rate values in diabetic mice treated with abatacept or vehicle show no statistically significant differences between abatacept-administered group and vehicle group. B) Delta urinary albumin excretion rate from baseline. C) Urinary albumin creatinine ratio values show no statistically significant differences between abatacept-administered group and vehicle group. Data are presented as mean±SEM * p<0.05, ** p<0.01 *** p<0.001 for difference to the abatacept group using One-way ANOVA. A non-diabetic control group is included for reference. D) Periodic acid–Schiff (PAS) staining of longitudinal cross-sectioned kidney was performed to investigate the effect of abatacept on mesangial expansion (ME). Mice were sacrificed at 14 weeks following treatment with streptozotocin. Fraction of all glomeruli affected by mesangial expansion shown as percentage (%) of glomeruli with ME. Data are presented as mean±SEM *** p<0.0001 for difference between non-diabetic and diabetic vehicle groups using One-way ANOVA. No statistically significant differences between abatacept-administered group and vehicle group. (E) Representative PAS stain of non-diabetic vehicle, diabetic vehicle and abatacept-administred group.

Albumin excretion rate (Fig 1A and 1B) and albumin/creatine ratio (Fig 1C) increased between 6 and 10 weeks following treatment with streptozotocin indicating the onset of diabetic nephropathy. Albuminuria progressed to expected levels [11] and remained unaffected by treatment with abatacept or vehicle (Fig 1).

Periodic acid–Schiff (PAS) staining of longitudinal cross-sectioned kidney was performed to investigate the effect of abatacept on mesangial expansion. 14 weeks following treatment with streptozotocin, the glomeruli of the diabetic mice showed mild mesangial expansion. Abatacept did not affect the development of mesangial expansion in the streptozotocin-induced mouse model of diabetic nephropathy (Fig 1D and 1E).

**Fig 2:**
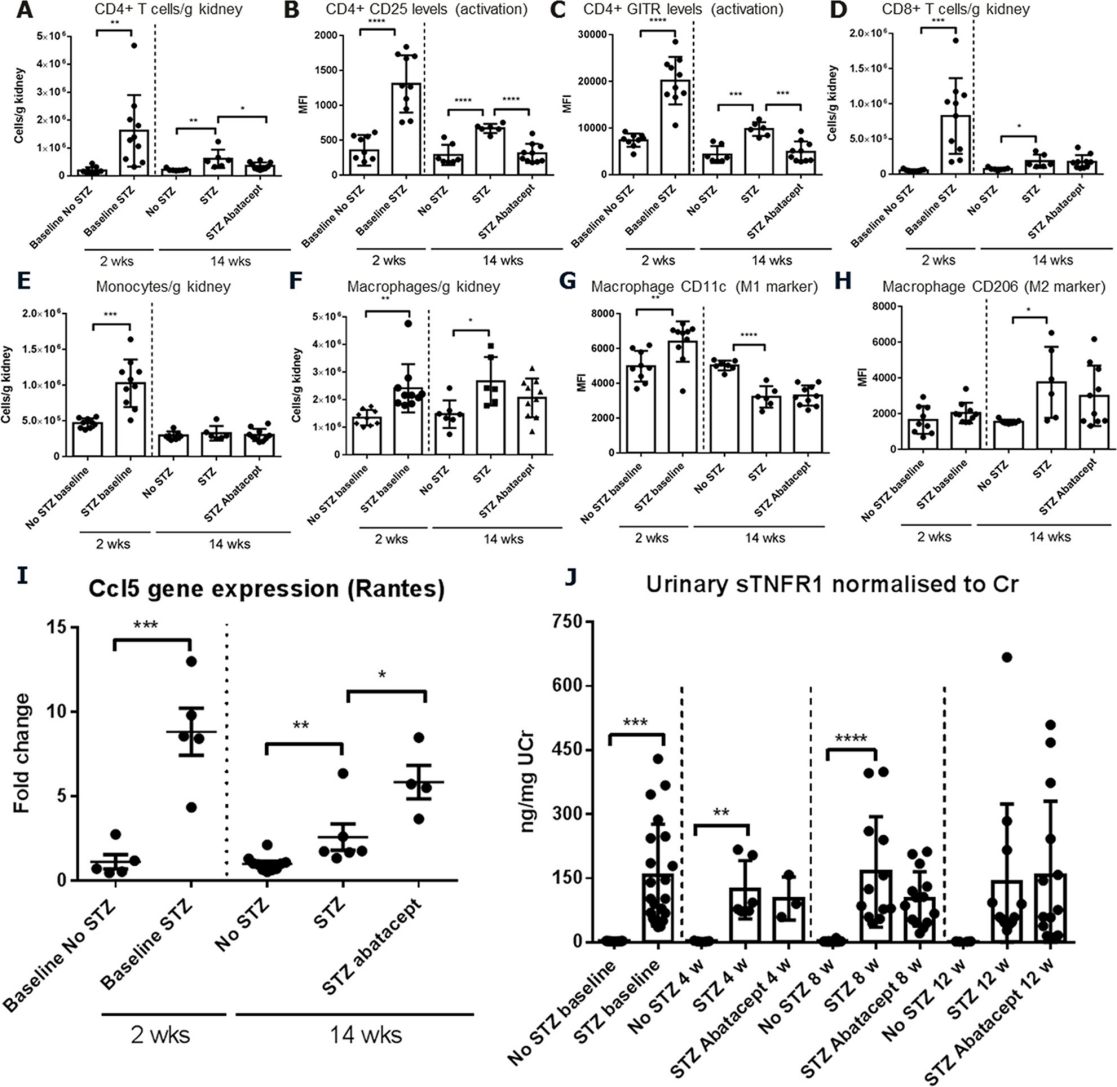
Effect of abatacept on kidney cellular infiltrates, kidney Rantes gene expression and the urinary biomarker sTNFR1. Kidney leukocytes were quantified by flow cytometry and cell number per tissue weight or mean fluorescence intensity for specific surface markers are indicated. A) Quantification of kidney CD4^+^ T cells. Kidney CD4^+^ T cells were increased in diabetic mice 2 and 14 weeks post STZ treatment, and were reduced by abatacept treatment. B) CD25 and C) GITR surface expression levels on CD4^+^ T cells. CD25 and GITR expression was increased in diabetic mice and reduced by abatacept treatment. D) Quantification of kidney CD8^+^ T cells. Kidney CD8^+^ T cells were increased in diabetic mice 2 and 14 weeks post STZ treatment, and were unaffected by abatacept treatment. E) Kidney monocytes were increased in diabetic mice 2 weeks post STZ treatment, but not 14 weeks after STZ treatment and monocytes were not influenced by abatacept treatment. F) Quantification of kidney macrophages. Kidney macrophages were increased in diabetic mice 2 and 14 weeks post STZ treatment, and numbers were not changed abatacept treatment. G) Kidney macrophage CD11 surface expression was reduced in diabetic mice and unaffected by abatacept treatment. H) Kidney macrophage intracellular CD206 (MR) expression was increased in diabetic mice and not changed by abatacept treatment. Baseline groups were compared by student’s t-test and treatment groups were compared by 1-way ANOVA and Sidak's multiple comparisons test. p<0.05: *; p<0.01: **; p<0.001: ***; p<0.0001: ****. I) Kidney gene expression was measured by qPCR. One gene (Ccl5, Rantes) of the ten genes measured (S1 Table) was significantly changed by abatacept treatment. Rantes gene expression was increased 2.3-fold in abatacept treated diabetic mice compared to vehicle-treated diabetic mice and 5-fold compared to non-diabetic mice. Baseline groups were compared by student’s t-test and treatment groups were compared by 1-way ANOVA and Sidak's multiple comparisons test. p<0.05: *; p<0.01: **; p<0.001: ***. J) The diabetic nephropathy progression biomarker sTNFR1 was measured in urine and levels normalised to urinary creatinine. Urinary sTNFR1 was significantly increased in urine from diabetic mice before treatment commenced, and at 4 and 8 weeks of the treatment period (2, 6 and 10 weeks after STZ treatment, respectively). Abatacept treatment did not affect the sTNFR1 levels in the urine. p<0.01: **; p<0.001: ***; p<0.0001: **** (student’s t-test diabetic vs. non-diabetic mice).

The kidney of acutely diabetic animals (2 weeks post STZ) showed increased leukocyte presence compared to non-diabetic mice as measured by flow cytometry (Fig 2). The kidney infiltrate was composed mainly of monocytes, macrophages and T lymphocytes, comprising both CD4^+^ and CD8^+^ T cells. The number of T cells and monocytes in the infiltrate was lower at 14 weeks than at 2 weeks after STZ treatment, indicating that the peak of immune cell recruitment occurs at the earlier time point. Importantly, kidney infiltration of CD4^+^ cells, CD4^+^CD25^+^, and CD4^+^GITR^+^ cells was reduced following abatacept treatment (Fig 2A–2C), which serves as a pharmacodynamic surrogate marker for the effect of costimulation blockade on T cell recruitment to the kidney. In keeping with CTLA-4Ig-mediated co-stimulation blockade acting exclusively on T cells, macrophage and monocyte parameters were not affected by treatment (Fig 2E–2H).

To assess whether expression of nephropathy-related mediators involved in renal function and albumin excretion was altered following treatment, we determined their mRNA levels in the kidney. A panel of 10 gene expression endpoints had previously been established in the STZ-induced mouse model of diabetic nephropathy, reflecting early fibrotic changes, inflammation and kidney injury. Abatacept treatment did not affect the majority of qPCR parameters tested in this model (S1 Table). Only the expression of CCL5 (a chemokine essential for creating haptotactic gradients to guide the migration of leukocytes into inflammatory sites [12]) was up-regulated following treatment with abatacept (Fig 2I).

The effect of abatacept on the concentration of the urinary biomarker sTNFR1, normalized to the amount of creatine, was investigated before and 4, 8 and 12 weeks after the start of treatment. Streptozotocin treatment increased the urinary levels of this biomarker, as expected [13] but abatacept treatment did not alter the urinary sTNFR1 content (Fig 2J).

**Fig 3:**
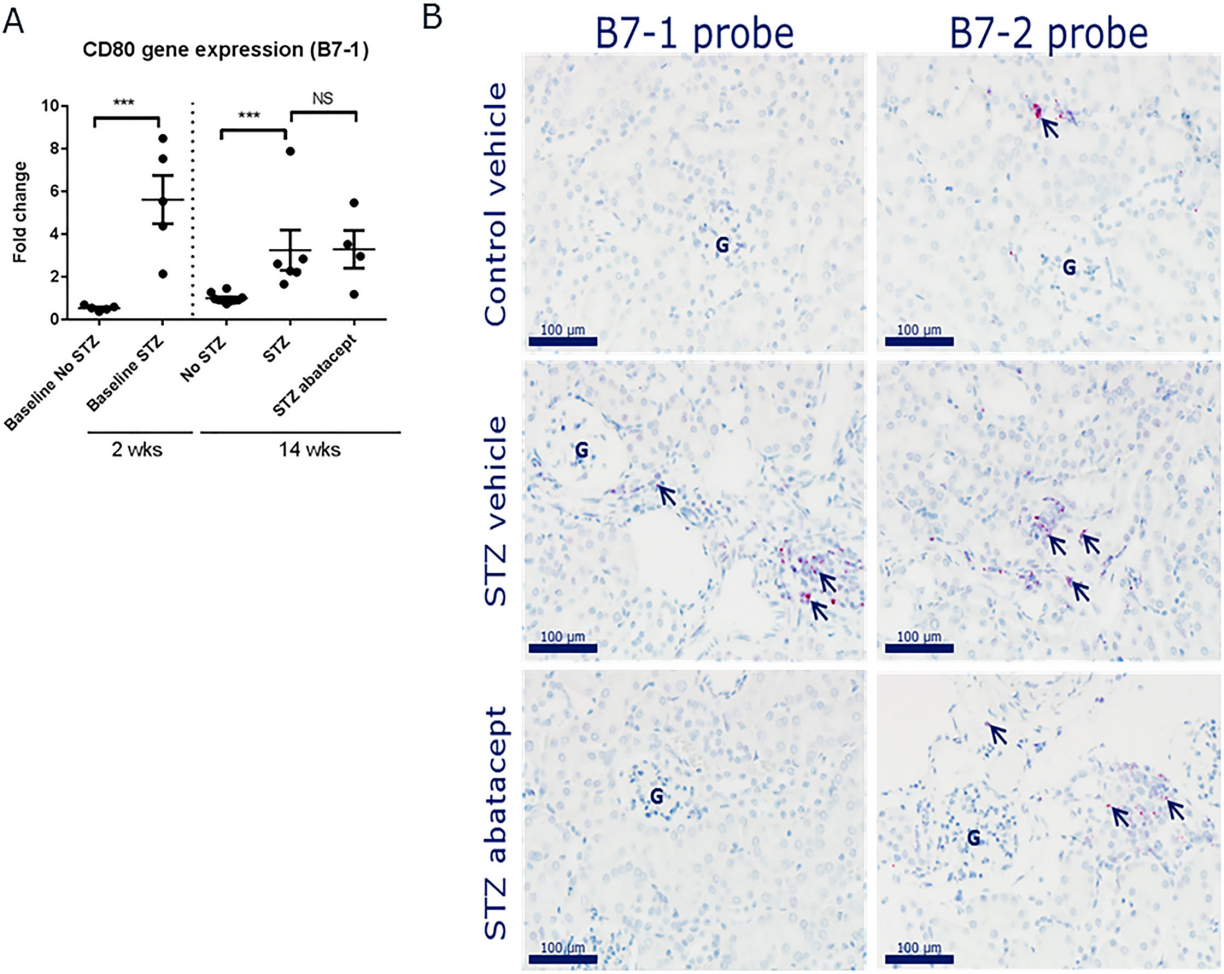
B7-1 and B7-2 mRNA expression in kidneys from STZ–diabetic mice treated with abatacept. A) Quantitative real-time PCR data for B7-1 in kidney tissues from treated animals and controls sampled at 2 and 14 weeks after STZ administration. B) *In situ* expression analyses of B7-1 and B7-2 mRNA expression in paraffin-embedded kidneys from control-vehicle, STZ-vehicle and STZ-abatacept treated mice (n = 5 per group).

Finally, although abatacept had no renoprotective effects in STZ mice, we evaluated whether B7-1 expression levels were altered in kidney samples from control and diabetic mice, treated or not with abatacept. First, quantitative assessment of renal B7-1 levels was performed via quantitative real-time PCR. An increase was observed after STZ treatment, both after 2 and 14 weeks (Fig 3A). Abatacept treatment, however, did not affect these levels.

This expression pattern resembles the gene expression profiles of the monocyte/macrophage-derived markers measured in S1 Table and Fig 2I, showing increased renal expression after STZ. We therefore hypothesized that B7-1 (and B7-2) expression localizes to infiltrating cells, and decided to test this by in-situ hybridization (ISH). Strong signals for B7-1 and B7-2 mRNA transcripts were observed in inflammatory aggregates in a kidney with active-chronic inflammation (S1 Methods). The expression level of B7-1 tended to be lower than that of B7-2. B7-1 and B7-2 mRNA transcripts were detected in neither glomeruli nor in tubular epithelial cells. In kidneys from diabetic and non-diabetic mice without active-chronic inflammation, the level of B7-1 and B7-2 mRNA transcripts was markedly lower and detected only in smaller perivascular immune cell aggregates and in a few scattered tubulo-interstitial cells (Fig 3B). Again, B7-1 and B7-2 mRNA transcripts were detected in neither glomeruli nor in tubular epithelial cells.

Taken together, these data indicate that B7-1 (and B7-2) is not expressed on podocytes neither in healthy nor diabetic animals. Instead, the expression pattern observed here is consistent with that of infiltrating immune cells.

## Discussion

The observation that B7-1 is expressed on podocytes in DN and that CTLA4-Ig therapy reduces albuminuria in animal models for DN raised the possibility that abatacept could be used as a treatment for patients with DN [4]. Our current data contradict this hypothesis by showing that, in the STZ model, B7-1 is not expressed on podocytes and abatacept treatment does not alter any of the DN parameters studied. This is in agreement with the recent study by Gagliardini et al, which failed to demonstrate B7-1 expression in kidneys from diabetic STZ mice [5].

Differences in mouse strain, STZ diabetes induction regimen and abatacept treatment protocol could account for some divergence between our present data and Fiorina et al. Fiorina et al waited for the mice to become hyperglycemic (2–4 days) to start CTLA4-Ig dosing. We instead decided to initiate dosing 18 days after STZ for the following reasons. First, we intended to ensure that the animals were truly diabetic prior to treatment initiation. With the multiple dose STZ protocol it takes around two weeks for the animals to consistently develop a stable state of hyperglycemia. If treatment is initiated earlier, it may interfere with beta cell destruction and diabetes development. Given the known immune component in the STZ model, CTLA-4Ig treatment could easily be envisioned to interfere. In addition, we found that around 2 weeks post STZ, there was an increased presence of leukocytes in the kidney tissue (Fig 2). This time point is therefore relevant in order to study the influence of T cell costimulation blockade on nephropathy progression. Collectively, we initiated treatment at a more advanced stage of disease progression and at the time when the target cell subset of CTLA-4Ig is present in the kidney. We argue that this is the most optimal timing from a translational clinical perspective. The CTLA-4Ig dose regimen was based on that used by Fiorina et al, who used 250 micrograms which translates to 10mg/kg for a 25 gram mouse, which is the dose we used. According to public EMA files the elimination half-life of abatacept in mice is approximately 3 to 6 days. In separate studies using a monoclonal antibody we determined that there is no difference in t½ in mice with diabetic nephropathy compared to healthy mice. Thus, hyperfiltration in diabetic mice does not affect plasma levels of antibody-sized compounds. We therefore expected to attain sufficient exposure, comparable to the Fiorina et al study, using 10 mg/kg 3xweekly.

Despite the above differences in treatment regimen, our unequivocally negative results with regard to DN amelioration indicate that the B7-1 pathway, be it podocyte or immune mediated, is unlikely to constitute a pivotal axis in the pathology of this model.

Whereas the role of podocyte-mediated B7-1 signalling in DN was called into question, the possibility still existed that CTLA4-Ig would exert beneficial effects through immune mediated pathways. We were able to demonstrate that abatacept therapy significantly decreases the accumulation of activated CD4^+^ T cells in the kidneys. Given the lack of correlation with DN outcomes, we conclude that T cell recruitment to the kidneys may not be a driving factor in the pathology of this model. CCL5 is produced by a wide array of immune and non-immune cells and is broadly chemoattractive for T lymphocytes, monocytes, natural killer cells, basophils and eosinophils, and can also activate immune cells [12]. Previous data showed upregulation of CCL5 mainly in tubular epithelial cells of patients with DN, suggesting involvement in the disease pathogenesis, possibly through macrophage/monocyte and lymphocyte recruitment and activation [14]. Although speculative, its upregulation observed here in the kidneys of abatacept treated STZ mice, may be part of a compensatory feedback mechanism in response to lowered T cell influx.

It is acknowledged that no current model adequately reproduces the pathophysiology of DN, in particular the late stages [11]. However, in conjunction with the recent data discounting the expression of B7-1 on podocytes [5], our present findings do not support a role for abatacept in DN treatment. Finally, we found that therapeutic reduction of activated T cell influx in the kidneys does not correlate with disease progression, which suggests that T cell inflammation is not a pivotal disease component.

## Concise methods

### Mice

Sv129 male mice (6–8 weeks old) were purchased from Charles River (Sulzfeld, Germany) and housed in groups of 10 mice according to Animal Unit’s SOP (Housing of experimental animals at Novo Nordisk A/S) with a 12-hour light/dark cycle. All mice were fed standard rodent chow (Altromin 1324, Brogaarden, Lynge, Denmark) and water ad libitum. Principles of laboratory animal care were followed and study approval was obtained from the Animal Experiments Inspectorate, Danish Ministry of Justice. Approval reference: BidR 2012/561-166; Dyreforsøgstilsynet, Slotsholmsgade 10, 1216 København K, Denmark. If visible signs of toxic effect were to be observed during the experiment, the mice were to be sacrificed immediately. The animals were weighed 3x weekly and if an individual mouse had lost 20% of body weight, treatment was to be started with 2x100μl 0.9% saline subcutaneous water or a single dose of insulin at 80 nmol/kg subcutaneously 3x week followed by observation. If the weight loss progressed below 20% then the animal was to be sacrificed. Termination of animals: The mice were weighed before anaesthesia with isoflourane. The animals were euthanised by perfusion with heparin saline.

### Treatment

Mice (8–10 weeks of age, >23g) were fasted for 4 h, then injected intraperitoneally with 125 mg/kg streptozotocin (made fresh in 0.05 M sodium citrate buffer, pH 4.5) or citrate buffer alone twice three days apart. Development of diabetes was defined by blood glucose >16 mmol/l in two consecutive measurements 1–2 weeks after the first streptozotocin injection. Mice in the diabetic groups with blood glucose levels below 16mmol/l (non-responders) or above 32mmol/l (uncontrolled responders) were not included in the study. The remaining 46 mice were randomized into two groups with equal blood glucose at the start of dosing as follows: diabetic vehicle (n = 23) and diabetic abatacept treated (n = 23). A non-diabetic vehicle group was added as an additional control group. Treatment with abatacept 10 mg/kg or appropriate control was given by subcutaneous injection three times a week from 2.5 weeks (18 days) after the first streptozotocin injection. The study was ended after hyperglycemia was established for at least 13 weeks. Mice were sacrificed by overdose of inhaled isoflurane followed by cervical dislocation.

### Assessment of diabetic nephropathy

The animals were weighed 3 times weekly on a digital scale and the abatacept dose adjusted to current body weight. Blood glucose was measured three times per week for the first two weeks and subsequently once per week on Biosen 5040. Blood glucose samples were taken in non-fasted mice before dosing. HbA1c% was assessed every three weeks starting one week after streptozotocin.treatment. HbA1c% was analysed on a Cobas 6000. At baseline and during weeks 6, 10 and 14 of diabetes the mice were placed individually for 16 hours in metabolic cages (Techniplast) for the collection of urine. The total amount of urine was weighed and a sample of 20 μL was taken for analysis of urine albumin and pre-diluted 20:380 with buffer containing 50 mM TBS pH 8.0, 1% BSA, 0.05% tween20. Samples were analysed using a sandwich ELISA from Bethyl Labs with some modifications. Upon sample collection the urine was diluted 20-fold in kit sample diluent prior to storage at -20°C. 24h albumin excretion rate was calculated from the 16h collection. Mouse creatinine was analysed with HPLC-UV from the same samples. One way ANOVA (multiple testing) was performed on albumin excretion rate and albumin/creatinine ratio.

### Histopathological evaluation of mesangial expansion

Periodic acid–Schiff (PAS) staining of longitudinal cross-sectioned kidney was performed to investigate the effect of abatacept on mesangial expansion.

### *In situ* hybridization for B7-1 and B7-2 mRNA expression in kidney samples

In situ expression analyses were performed using RNAscope in situ hybridization (ISH) kit from Advanced Cell Diagnostics. Probes for B7-1 and B7-2 mRNA were applied onto paraffin-embedded kidneys from control-vehicle, STZ-vehicle and STZ-abatacept treated mice (n = 5 per group). Positive and negative control probes (PPIB and DapB, respective) were used to qualify the tissue samples for use in the ISH application. A kidney with active-chronic inflammation from a STZ-vehicle mouse served as a positive control for detection of B7-1 and B7-2 mRNA expression (S1 Methods).

### Renal biomarker analysis

Kidneys were harvested from diabetic and control mice, RNA isolated (TriZOL followed by RNEasy, Qiagen) and gene expression analysed by TaqMan qPCR analysis (Life Technologies) following the manufacturer’s instructions, and employing as reference genes: 18S, Rpl27, Rps13, and Ubc. Unpaired t-tests were performed on log2 transformed fold change values. Kidney leukocyte content was assessed by flow cytometry. Briefly, total kidney cells were blocked for unspecific binding with anti-CD16/CD32 (BD Biosciences), followed by surface staining of CD4 and CD11c (BD Biosciences), CD8, CD25, GITR, CD11b, and F4/80 (BioLegend), CD45 (eBioscience), and CD206 (AbDSerotec). 7-AAD (BD Biosciences) was included as a dead cell marker. Samples were acquired on a FACS LSRFortessa followed by data analysis using FACSDiva software (BD Biosciences). Kidney leukocytes were identified as viable CD45^+^ cells. Kidney macrophages were identified as viable, CD45^+^ CD11b^+^ F4/80^+^ cells and monocytes as CD45^+^ CD11b^+^ F4/80^-^.

### Urinary biomarker analysis

sTNFR1 was measured in urine samples by Milliplex assay (MerckMillipore) according to the manufacturer’s instructions and normalised to urinary creatinine measured by HPLC.

### Statistical analysis

Graphpad Prism software was used and all statistical tests applied are detailed in the respective figure legends.

## Supporting Information

S1 Fig The effect of abatacept treatment on the development of diabetes in the streptozotocin-induced mouse model of diabetic nephropathy. (DOCX)

S1 Methods (DOCX)

S1 Table The effect of abatacept on gene expression endpoints (qPCR) in the streptozotocin-induced mouse model. (DOCX)
