# Supplementary material for: Abatacept Treatment Does Not Preserve Renal Function in the Streptozocin-Induced Model of Diabetic Nephropathy
Source: PLoS One. 2016 Apr 7;11(4):e0152315. doi: 10.1371/journal.pone.0152315 (PMC4824484; doi:10.1371/journal.pone.0152315)

**S1** **Fig. The effect of abatacept treatment on the development of diabetes in the streptozotocin-induced mouse model of diabetic nephropathy.** A non-diabetic control group is included for reference. a) Blood glucose in diabetic mice treated with abatacept or vehicle is significantly elevated compared to non-diabetic control group. p<0.001 for difference between abatacept-administered group and non-diabetic control group using two-way ANOVA. b) Body weight in diabetic mice treated with abatacept or vehicle is significantly reduced compared to non-diabetic control group. p<0.001 for difference between abatacept-administered group and non-diabetic control group using two-way ANOVA. c) Glycosylated HbA1c% in diabetic mice treated with abatacept or vehicle is significantly increased compared to non-diabetic control group. p<0.001 for difference between abatacept-administered group and non-diabetic control group using two-way ANOVA. Data are presented as mean±SEM.


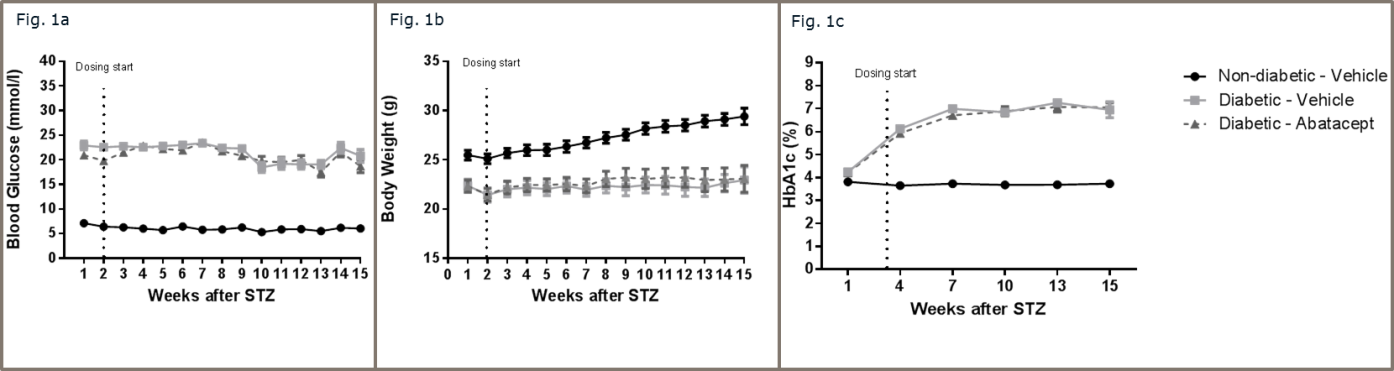

Supplement: S1 Fig — (DOCX) [file pone.0152315.s001.docx]
