# Supplementary material for: Abatacept Treatment Does Not Preserve Renal Function in the Streptozocin-Induced Model of Diabetic Nephropathy
Source: PLoS One. 2016 Apr 7;11(4):e0152315. doi: 10.1371/journal.pone.0152315 (PMC4824484; doi:10.1371/journal.pone.0152315)

**S1 Methods**

Preparation of abatacept dosing solution: The abatacept (Orencia) 250 mg powder was diluted with 0.9% NaCl to a dosing solution of 2 mg/mL and frozen in aliquots. Each dose was freshly thawed at room temperature before dosing i.p.

Blood glucose: Blood samples were obtained from the tip of the tail by puncturing the tail vein with a lancet. 5μl of blood was collected into heparinised capillary tubes and transferred to 250μl glucose buffer (Glucose/lactate system solution, EKF Diagnostics, Barleben, Germany) for blood glucose measurements. The samples were stored at room temperature until specimens had been sampled from all mice and were analysed on the same day using a Biosen5040 Glucose Analyzer (EKF Diagnostics, Barleben, Germany)

HbA1c: 5μl of blood was collected into a heparinised capillary tube and was transferred to 500μl assay buffer (HbA1c Hemolyzing Reagent for Tina‑quant HbA1c, EKF Diagnostics, Barleben, Germany). The samples were stored at -20⁰C until analysed using a Cobas HbA1c Analyser (Roche, Rotkreuz, Switzerland).

Mouse urine albumin was measured using a sandwich ELISA from Bethyl Labs (Cat. no. E90-134) with some modifications. Upon sample collection the urine was diluted 20-fold in kit sample diluent prior to storage at -20°C. The plates were coated with capture antibody by adding 100 µl to the wells and incubating over night at 4°C. The plates were washed according to protocol and blocked with 200 µl blocking buffer for at least 30 min. If not used immediately they can be sealed and stored at 4°C for up to 4 days without loss of signal. Calibrators with mouse albumin were prepared in sample diluent at the following concentrations, 6000, 2857, 1361, 648, 309, 147, 70, and 0 ng/ml. The blocked plates were washed and 100 µl sample diluent was added followed by 2 µl of sample/calibrator. Calibrators were added in duplicates. The plates were incubated 2 hours at RT with shaking followed by washing, and were subsequently incubated one more hour with 100 µl HRP labelled detection antibody. After washing the plates were incubated for 20 minutes with 100 µl TMB solution before the reaction was stopped with 100 µl stop solution. Absorbance was read at 460/620 nm using a BioTek microplate reader. Data was analysed with a LOCI Calculator, which offers a graphical user interface to the R environment for statistical computing (www.r-project.org). Standard curves were fitted using a 5-parameter logistic model with a 1/Y2 weighing function. The data exclusion criteria were determined by the lower limit of quantification (LLOQ), 50 ng/ml and the highest calibrator concentration, 6000 ng/ml. The LLOQ is the highest value at which the calculated %CV is less than 20% and the recovery of each calibrator is within 80-120% of the known value. Each assay plate also includes three controls in duplicates at 5000, 600, and 110 ng/ml. The plate is accepted if at least four out of the six controls are within ±20% of the expected value.

Mouse urinary creatinine was measured with HPLC-UV (Accela). 25 µl mouse urine was transferred to eppendorf tubes, to which 250 µl acetonitrile with 0.5 % v/v acetic acid was added. After vortexing and centifugation at 13000 rpm for 20 min at 4 degrees Celcius. 250 µl from each sample was transferred to a Greiner plate and allowed to evaporate at 40 degrees Celcius for 45 minutes. The samples were redissolved in 65 µl Na-acetate (5 mM pH 4.1) for 20 min with shaking and 60 µl volume from each sample was subsequently transferred to a new Greiner plate and analysed on HPLC-UV at 225 and 236 nm.

Flow cytometry: Kidneys were harvested from diabetic abatacept- and vehicle treated and non-diabetic vehicle-treated mice 14 weeks after STZ-treatment and kept in Belzer Cold storage solution (Bridge of Life, Columbia, SC) at 5°C until analysis the following day. Kidneys from a separate group of diabetic and non-diabetic mice were collected 2 weeks after STZ-treatment, corresponding to initiation of treatment in the treated mice.

Kidneys were cut into small pieces and digested in media (RPMI with 5% serum) supplemented with 2 mg/ml collagenase II (Gibco) and 50 U/ml DNase (Sigma) by incubating for 40 min at 37°C on a roller mixer. To release glomeruli from interstitium the samples were mixed by pipetting before passing through a 100 μm cell strainer (BD Biosciences). The cell suspension was washed and erythrocytes were lysed with RBC lysis buffer (eBioscience). To digest glomeruli, the preparation was further incubated in media supplemented with 0.5 mg/ml collagenase type II, 50 mg/ml dispase II (Sigma), 50 U/ml DNase and 0.075% trypsin (Gibco). The cells obtained were washed, resuspended in 1x HBSS without salts with 2 mM EDTA and kept for 10 min on ice. In order to disrupt tubular structures the suspension was passed 3 times through a needle (23G). The cells were finally passed through a 40 μm cell strainer (BD Bioscience).

Flow cytometric analysis was performed according to standard procedures. Briefly, cells were blocked for unspecific binding with anti-CD16/CD32 (BD Biosciences), followed by surface staining of CD4 and CD11c (BD Biosciences), CD8, CD25, GITR, CD11b, and F4/80 (BioLegend), CD45 (eBioscience), and CD206 (AbDSerotec). 7-AAD (BD Biosciences) was included as a dead cell marker. Samples were acquired on a FACS LSRFortessa followed by data analysis using FACSDiva software (BD Biosciences). Kidney leukocytes were identified as viable CD45^+^ cells. Kidney macrophages were identified as viable, CD45^+^ CD11b^+^ F4/80^+^ cells and monocytes as CD45^+^ CD11b^+^ F4/80^-^.

qPCR: Kidneys were harvested from diabetic and control mice at study termination and kept frozen at –80°C until analysis. Kidney samples were lysed in TriZOL (Life Technologies) and RNA isolated using the RNeasy mini kit incl. DNase treatment (Qiagen). cDNA was generated using the High Capacity cDNA kit (Life Technologies). cDNA was loaded in 384-well microplate Taqman arrays using Fast mastermix (Life Technologies). qPCR was performed on the QuantStudio system (Life Technologies). Data were analysed by the ΔΔCt method, first normalising gene expression of each target gene to the average of four reference genes (18S, Rpl27, Rps13, Ubc),and next normalising gene expression of each target to the average of the non-diabetic group. Unpaired t-tests were performed on log2 transformed fold change values.

Histopathology on kidneys: At study termination, mice were sacrificed and the kidneys were sampled and processed by standard histological procedures. Briefly, the right kidney was fixed in 4% paraformaldehyde in PBS for 24 hours and transferred to 70% ethanol. The kidney samples were processed in a Leica Asp300S histoprocessor (Leica Microsystems, Ballerup, Denmark) overnight and embedded in paraffin blocks using a Shandon Histocentre 3 (Thermo Electron Corporation, Marietta, Ohio). Two μm thick paraffin sections were cut of the kidney samples, mounted onto Superfrost Plus slides (Fisher Scientific), dried for 1 hour at 60°C and stored at 4°C until use. Deparaffinised and rehydrated sections were stained with periodic-acid schiff´s reagent (PAS) (Merck cat. no. 1.00524 and Sigma cat. no. 3952016, respectively), dehydrated and mounted in Pertex (Histolab Products cat. no. 00801). The histopathology of kidneys was examined in an Olympus AX70 microscope in a blinded fashion using the following criteria: The frequency of glomeruli containing deposition of homogenous PAS positive material/mesangial expansion was assessed from one PAS stained kidney section from each animal. Values are mentioned as mean ± SEM.

In situ hybridization for B7-1 and B7-2 mRNA expression in kidney samples: kidneys with active-chronic inflammation from a STZ-vehicle mouse served as a positive control for detection of B7-1 and B7-2 mRNA expression.


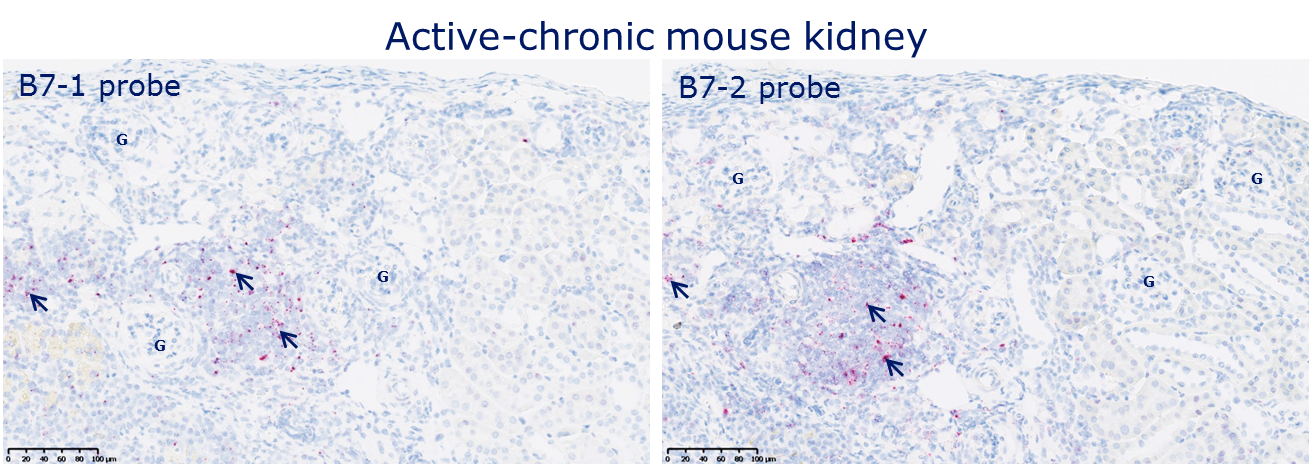

Supplement: S1 Methods — (DOCX) [file pone.0152315.s002.docx]
