# Supplementary material for: Abatacept Treatment Does Not Preserve Renal Function in the Streptozocin-Induced Model of Diabetic Nephropathy
Source: PLoS One. 2016 Apr 7;11(4):e0152315. doi: 10.1371/journal.pone.0152315 (PMC4824484; doi:10.1371/journal.pone.0152315)
Supplement: S1 Table — (DOCX) [file pone.0152315.s003.docx]

**S1 Table. The effect of abatacept on gene expression endpoints (qPCR) in the streptozotocin-induced mouse model**

Kidney gene expression was measured by Taqman qPCR. Fold changes for diabetic mice vs. non-diabetic mice were calculated at treatment start (baseline group terminated) and at study termination. Fold change for the abatacept treated group vs. the diabetic group is indicated.

| Gene | Role | STZ vs. No STZ  2 weeks  Fold change (p-value, t-test) | STZ vs. No STZ  14 weeks  Fold change (p-value, t-test) | STZ abatacept vs. STZ  14 weeks  Fold change (p-value, t-test) |  |
| --- | --- | --- | --- | --- | --- |
| Ccl2 (MCP-1) | Inflammation | 13.6 (2.72E-06) | 6.0 (2.37E-07) | 1.3 (0.2393) |  |
| Ccl5 (Rantes) | Inflammation | 7.8 (0.0003) | 2.6 (0.0031) | 2.3 (0.0188) | |
| Fibronectin | Fibrosis | 1.8 (0.0024) | 1.9 (0.0053) | 1.2 (0.4099) |  |
| HAVCR1 (KIM-1) | Injury marker | 1.5 (0.0228) | 1.3 (0.1011) | 1.1 (0.5091) |  |
| ICAM1 | Endothelial inflammation | 2.8 (0.0002) | 1.9 (0.0007) | 0.9 (0.7591) |  |
| LCN2 (NGAL) | Injury marker | 5.3 (4.75E-05) | 3.0 (0.0003) | 1.6 (0.1448) |  |
| Serpine1 (PAI-1) | Fibrosis | 2.0 (0.0002) | 3.8 (0.0198) | 0.7 (0.9767) | |
| TGFb1 | Fibrosis | 2.5 (0.0007) | 1.4 (0.0470) | 1.2 (0.2407) |  |
| TNF | Inflammation | 8.4 (1.47E-06) | 3.5 (0.0012) | 1.3 (0.4988) |  |
| VCAM1 | Endothelial inflammation | 7.8 (8.26E-06) | 3.3 (1.90E-05) | 1.3 (0.3243) |  |
